# Protein Supplementation and Grazing Behavior for Cows on Differing Late-Season Rangeland Grazing Systems

**DOI:** 10.3390/ani11113219

**Published:** 2021-11-11

**Authors:** James E. Sprinkle, Joseph K. Sagers, John B. Hall, Melinda J. Ellison, Joel V. Yelich, Jameson R. Brennan, Joshua B. Taylor, James B. Lamb

**Affiliations:** 1 Nancy M. Cummings Research, Extension & Education Center, University of Idaho, Carmen, ID 83462, USA; jbhall@uidaho.edu (J.B.H.); ellison@uidaho.edu (M.J.E.); yelich@uidaho.edu (J.V.Y.); 2 Jefferson & Clark County Extension, University of Idaho, Rigby, ID 83442, USA; jsagers@uidaho.edu; 3 West River Research & Extension Center, South Dakota State University, Rapid City, SD 57702, USA; jameson.brennan@sdstate.edu; 4 U. S. Sheep Experiment Station, USDA Agricultural Research Service, Dubois, ID 83423, USA; bret.taylor@ars.usda.gov; 5 Formerly Furst-McNess Company, Currently Intermountain Farmers Association, Rexburg, ID 83440, USA; buckwt@gmail.com

**Keywords:** accelerometer, beef cattle, grazing behavior, grazing systems, protein supplementation, residual feed intake

## Abstract

**Simple Summary:**

Cattle grazing late-season dormant rangeland are subject to impaired production due to reduced forage digestibility and a longer residence time of forage in the rumen, leading to reduced forage intake. It is a common practice to provide supplemental protein to help counteract these effects and to improve animal well-being and livestock production. Yet, the usage of supplements has been shown to interrupt and reduce the time spent grazing. These behavioral changes may vary with climate and the frequency and timing of strategic supplementation. The objective of this study was to evaluate how protein supplementation altered grazing behavior when used in both rotationally and continuously grazed dormant pastures. We utilized accelerometers (used in rockets to measure velocity in three directions and in smart phones to rotate the screen) to evaluate cattle behavior (via head movements) every 5 s on a 24 h basis. The cattle altered their grazing behavior in response to climate, supplementation status, and the grazing system. Cattle that were deprived of the protein supplement and stayed in the same continuously grazed pasture showed more restlessness in their behavior, spending more time walking from midnight to 8 a.m. Additionally, the harvest rate of dormant forage increased for the supplemented cattle.

**Abstract:**

The objective was to determine if low- or high-residual feed intake (LRFI or HRFI, *n* = 24 for each) Hereford × Angus cows on continuously or rotationally grazed rangeland altered their grazing behavior when provided a protein supplement in late autumn. Treatments included continuously grazed, control (CCON, *n* = 12); continuously grazed, supplemented (CTRT, *n* = 12); rotationally grazed, control (RCON, *n* = 12); and rotationally grazed, supplemented pastures (RTRT, *n* = 12). Cows in each treatment had grazing time (GT), resting time (RT), and walking time (WLK) measured for 2 years with accelerometers. Bite rate (BR) was also measured. Time distributions of GT and RT differed by year (*p* < 0.05), being influenced by colder temperatures in 2016. Cattle in 2016 spent more time grazing during early morning and late evening (*p* < 0.05) and rested more during the day (*p* < 0.05). In 2017, cattle in the CCON treatment walked more (*p* < 0.05) during early morning time periods than did the CTRT cattle, indicative of search grazing. All supplemented cattle had greater BR (*p* < 0.05) than control cattle in 2017. Cattle with increased nutritional demands alter grazing behavior in a compensatory fashion when grazing late-season rangelands.

## 1. Introduction

Cows maintained on late-season rangeland in the Pacific Northwest of the USA often experience declining forage quality [1], which may fail to meet the protein requirements (7% of dry matter) necessary for adequate rumen function [2,3]. It is a common practice to supply additional protein to cattle grazing late-season rangeland. Yet, protein supplementation has been shown to decrease daily grazing activity, even when done as infrequently as once every 6 days [4]. 

Intuition would suggest that cattle maintained on a rotational grazing system may have an opportunity to select a higher quality diet than cattle which stay in the same pasture for the duration of the grazing period; however, past research conducted or reported by scientists in several locales [5,6,7] fail to support this conclusion. 

Extensive research [8,9,10] has been performed to classify beef cattle for their overall feed efficiency. Using specialized equipment (GrowSafe Systems, Ltd., Airdrie, AB, Canada), cattle can be compared with respect to residual feed intake (RFI), which is expressed as the difference between expected feed intake (based upon body weight and growth) and actual feed intake [11]. Cattle with negative RFI scores (low RFI: LRFI) consume less feed than expected and are classified as more efficient while cattle with positive RFI scores (high RFI: HRFI) consume more feed than expected and are classified as inefficient. Industry has embraced the adoption of using RFI data for bull purchases. Unfortunately, most research with RFI has been done in the feedlot, with minimal research being conducted in a grazing environment [12,13,14,15,16,17,18] and even less in a rangeland environment [19,20,21,22]. A key finding of our prior research [21] conducted on late-season, low-quality rangeland with unsupplemented nonlactating 2-year-old cows was that LRFI cows lost less weight and body condition than did HRFI cows; however, we did not observe any differences in either daily travel distance or harvesting rate (bite rate) between HRFI and LRFI cattle.

The objective of this study was to determine if 2-year-old cattle on either a continuous or rotational late-season rangeland grazing system altered the daily pattern of grazing, depending upon supplementation status or pasture treatment. Cow performance data (body weight) for these different supplementation strategies have been reported previously (23 conference proceedings) as well as gross measurements of the total hours of daily grazing, resting, and walking [23]. This study was also preceded by research to determine the feasibility of using accelerometers to determine grazing behavior in an extensive rangeland environment [24]. Both of these previously published studies were conducted at the same location as this study and used the same experimental cattle. We hypothesized that: (1) cattle with greater nutritional demands (HRFI cattle; non-supplemented cattle) would spend more time foraging, possibly reducing resting time and increasing grazing and walking time; (2) the pattern of daily grazing would differ as climatic conditions or nutritional status changed; and (3) harvesting efficiency would be altered as cattle experienced increasing nutritional deficits. 

The previous research conducted at this site established that cows differing for either nutritional status or metabolic feed efficiency altered their total daily activity (hypothesis 1; [23]) but failed to consider *how* the pattern of daily foraging behavior changed over the 24 h time period (hypothesis 2) or *how* forage harvesting efficiency would be affected for supplemented vs. non-supplemented cattle on different pasture treatments (hypothesis 3). In this research, we will describe some of the mechanistic adaptations that cattle pursue in limited grazing environments in an attempt to accommodate nutritional deficiencies.

## 2. Materials and Methods

### 2.1. Treatment Allocation

Materials and methods in this study are very similar to what we reported previously [23,24] but are duplicated again to enhance readability of this research. Collared cattle (*n* = 48) used in this two-year study were part of a larger group of cattle used to determine livestock production responses to protein supplementation. These cohort groups (*n* = 24) were different for 2016 and 2017. Two-year-old Hereford × Angus collared cattle were selected from a pool of replacement females that had been previously classified as either LRFI or HRFI as yearling heifers as described by Hall et al. [25] while being fed an 80% roughage diet. Heifers classified as LRFI had standard deviations ≤ 0.5 below the mean and those classified as HRFI had standard deviations ≥ 0.5 above the mean. 

In 2016 and 2017, Hereford × Angus cattle in the entire cowherd were allocated to 1 of 4 treatments: (1) grazed same pasture continuously with no protein supplementation (CCON, *n* = 75); (2) grazed same pasture continuously with 3.17 kg Bova Cubes (28% crude protein dried distillers grain protein cube, Furst–McNess Company, Freeport, IL, USA, Table 1) per cow fed once a week (CTRT, *n* = 71); (3) grazed rotated pastures (13 pastures in 2016, 2 pastures in 2017) with no protein supplementation (RCON, *n* = 73); or 4) grazed rotated pastures (13 pastures in 2016, 2 pastures in 2017) with 3.17 kg Bova Cubes fed as described above (RTRT, *n* = 73). Cattle were randomized each year of the study to maintain similar beginning weights, body condition score (1 to 9, 9 = fattest), and average age for each treatment group. The supplemented cows were fed every Thursday (2016) or Friday (2017) at approximately 1200 h. The supplement was fed a total of six times in 2016, commencing on 2 November, and seven times in 2017, commencing on 27 October. In 2017, the cows were trained to respond to an audible cue (truck horn) when supplement was fed.

In 2016, 2-year-old cows (12 LRFI, 12 HRFI) were fitted with a homemade grazing halter [24] containing both a 3-axis accelerometer (USB Logger Model XB, Gulf Coast Data Concepts, LLC, Waveland, MS, USA) and a global positioning system (GPS) logger (iGotU GT-120, Mobile Action Technology, New Taipei City, Taiwan). Both the accelerometer and the GPS logger had an extended rechargeable Li-ion 3.7 V, 5200 mAh battery (Tenergy Li-ion 18650, Freemont, CA, USA) soldered to the equipment to extend data logging for up to 45 d with moderate ambient temperatures. The GPS results will not be reported in this manuscript.

Four of the cows carrying grazing halters were fitted with the halters on 24 October and the other 20 cows were outfitted on 18 October. There were 3 LRFI and 3 HRFI cows placed within each pasture treatment. Midway through the trial, on 17 November, all grazing halters were removed due to animal discomfort with the stiff nylon halters as the temperatures got colder. One of the cows had a halter removed on 7 November due to the halter being too tight and causing discomfort (HRFI cow on RTRT pasture). All grazing observation data from this cow were deleted. The accelerometer data on any day the cow was herded outside her experimental pasture to or from a corral for processing were excluded from data analyses. On 13 December, cows were shipped to the University of Idaho Nancy M. Cummings Research, Extension and Education Center at Carmen, Idaho (45°17.322′ N, 113°52.697′ W).

In 2017, 2-year-old cows were allocated to the same pasture treatments as described for 2016. The same equipment for the grazing halters was retrofitted to grazing collars [24] to eliminate sore noses from halters placed on cattle in late fall with stiffer nylon, longer winter hair, and less peripheral circulation. Grazing collars were left on the cows until they were shipped back to Carmen, ID on 12 December. Grazing behavior data were eliminated for all days when cattle were trailed to or from working corrals.

### 2.2. Range Sites

The trials were conducted from mid-October to mid-December in 2016 and 2017 at the U.S. Sheep Experiment Station (USSES), located about 16 km northeast of Dubois, Idaho (44°18′ N, 112°7′ W). In 2016, 44 CCON cows grazed a 359 ha pasture; 42 CTRT cows grazed a 1381 ha pasture; 25 RCON cows grazed 13 10 ha small pastures, moving every 3 to 4 d; and 25 RTRT cows also grazed similar 10 ha small pastures, moving every 3 to 4 d. In 2017, 31 CCON cows grazed a 90 ha pasture; 29 CTRT cows grazed a 79 ha pasture; 48 RCON cows grazed a 65 ha pasture and then another 65 ha RCON pasture, moving after 25 d; and 48 RTRT cows grazed a 65 ha pasture and then another 65 ha RTRT pasture, moving after 25 d. The rotational grazing treatments varied by year due to the availability of experimental pastures that could be used for this research trial. The much larger pastures used for CTRT and CCON treatments in 2016 had the water source (troughs) positioned near the edge of the pasture to reduce the zone of forage available to cattle.

The range sites were in the sagebrush steppe with elevations ranging from 1740 m to 1867 m in 2016 and from 1659 m to 1699 m in 2017. Slopes were generally less than 20% but mostly between 0% and 12%. The 20-year mean annual precipitation (1981 to 2010) near the research sites (44°15′ N, 112°12′ W, elevation, 1661 m) is 328 mm, with 58% falling during April through September. The pastures are dominated by mountain big sagebrush (*Artemisia tridentata* Nutt. ssp. *vaseyana* (Rydb.) Beetle) and threetip sagebrush (*A. tripartita* Rydb.) with subdominant shrub species including antelope bitterbrush (*Purshia tridentata* (Pursh) DC.), yellow rabbitbrush (*Chrysothamnus viscidiflorus* (Hook.) Nutt.), and spineless horsebrush (*Tetradymia canescens* DC.). Dominant perennial grasses included Great Basin wildrye (*Leymus cinereus* (Scribn. and Merr.) A. Löve), Idaho fescue (*Festuca idahoensis* Elmer), sandberg bluegrass (*Poa secunda* Presl), thickspike wheatgrass (*Elymus lanceolatus* (Scribn. and J.G. Sm.)), bluebunch wheatgrass (*Pseudoroegneria spicata* (Pursh) A. Löve), and needle and thread (*Hesperostipa comata* (Trin. and Rupr.) Barkworth), with only trace amounts of cheatgrass (*Bromus tectorum* L.). Common forbs included lupines (*Lupinus* spp. L.), milkvetches (*Astragalus* spp. L), fleabanes (*Erigeron* spp. L.), and pussytoes (*Antennaria* spp. Gaertn.). The soils within this site are predominantly loamy with numerous shallow soil areas, rocky soils, and exposed rock outcrops due to underlying volcanic deposits of basalt.

All sagebrush steppe pastures used in 2016 had a prior burn history. The 1381 ha CTRT pasture had been burned in 1998, 1999, 2003, 2008, and 2009 with a cumulative area burned of 38%, with existing estimated shrub cover of 38% and estimated sagebrush cover of 28%. The 359 ha CCON pasture had been burned in 2002 and 2008 with the cumulative area burned being 38%, estimated shrub cover being 36% and estimated sagebrush cover being 26%. The small, rotated pastures had been burned in the fall of 2008 and the spring of 2009, with the area burned averaging 68%, with a range from 53% to 95%. An estimated shrub cover for these pastures averaged 24% (range 28% to 32%) and the estimated sagebrush cover averaged 14% (range 8% to 21%).

In 2017, two of the sagebrush steppe pastures used had not been burned in 35 years (1st RCON pasture, and 2nd RTRT pasture) and the total shrub cover for both of these pastures was 21%. The remaining 4 pastures used in 2017 had been burned in 2000. The cumulative area burned averaged 54% and the total shrub cover averaged 12%.

### 2.3. Forage Production and Quality

In 2016, forage production was estimated at the beginning of the grazing period by hand clipping 10 randomized 0.16 m^2^ quadrats from both a site with a burn history and one without a burn history, from both continuously grazed pastures. Forage production for the 10 ha rotated pastures was estimated within a burned site using five randomized plots. All perennial and annual graminoids rooted within the quadrat frame within the sampled areas were clipped to ground level and dried for 24 h at 65 °C. Palatable half shrubs and edible forbs were clipped separately and analyzed as browse. The majority of the browse consisted of parsnip-flower buckwheat and only the current year’s plant leaders were clipped for this plant. Sagebrush canopy was not sampled for production. Forage production in 2017 was estimated in each pasture using the same procedures described above with 10 randomized plots. Sites chosen were representative of the pastures and were chosen in areas with some existing sagebrush canopy.

Crude protein [26,27,28,29] was determined on replicate samples (*n* = 5 clipped plots/replicate; (except for the 2016 grass sample for the rotated pasture and 2017 single browse sample in one pasture)) of clipped forage by a commercial lab (Ward Laboratories, Inc., Kearney, NB, USA). Forage digestibility of the clipped forage samples at the same lab was estimated in vitro from acid detergent fiber (ADF) using the ANKOM 200/220 Fiber Analyzer (ANKOM Co., Macedon, NY, USA) and following the procedures of Mertens [30].

### 2.4. Grazing Behavior Observations and Data Processing

Estimates of grazing time, resting time (including standing, lying down, and rumination), and walking time were estimated every 5 s using the 3-axis accelerometers described previously [24]. The accelerometers monitored head movement for 25 data points every s (25 hz) and these observations were averaged to every 5 s. These data were then summarized by day for each 2 h time period beginning at midnight [24]. 

Focal sampling for bite rate (BR, bites/min) was conducted on single animals [31] during either the a.m. or p.m. observation time periods for approximately 10 to 15 min. At least 4 replicate samples per observation period were acquired whenever possible. Beginning and ending times for each replicate were recorded in the field on a tablet computer using a spreadsheet with an integrated timestamp. Sometimes (8.3%) cattle commenced resting, walking to water, or ruminating in the midst of an observed grazing bout, so it was not always possible to obtain multiple sample replicates of 4 or greater during the grazing observation period. Bite rate frequency data were averaged over each observation period. 

There were 17 accelerometers that completed the full 29 d of data collection in 2016. One cow had data eliminated due to a sore nose (HRFI cow on RTRT pasture), and 6 accelerometers (days 10, 12, 14, 16, 19, and 20) shut down prior to d 29 due to loss of battery charge. In 2017, 12 accelerometers completed the full 45 days of data collection, 1 accelerometer malfunctioned (LRFI cow on CTRT pasture), 1 accelerometer was damaged by a cow (LRFI cow on RCON pasture), and the remainder of the accelerometers shut down prior to day 45 due to a loss of battery charge. The average battery life of the accelerometers in 2017 was 35 days. 

All days of complete data from all cows were used for data analysis with 547 days total in 2016 and 779 days total in 2017. The methodology for processing accelerometer data for all 2-year-old cows used in this trial is described more fully in [24].

### 2.5. Statistical Analyses

Daily grazing time, resting time, and walking time were analyzed using a restricted maximum likelihood-based mixed effects model for repeated measures (v. 9.4, SAS Inst., Inc., Cary, NC, USA) with the categorical, fixed effects of pasture treatment, year, RFI group and the interactions between RFI group × year and pasture treatment × year. Cow within (RFI group × pasture treatment) was included as a random effect and was the repeated subject. Bite rate was analyzed by year with the fixed effects of pasture treatment, day of bite rate determination, RFI group and the interaction between pasture treatment × day of bite rate and the same repeated, random subject. Nonsignificant interactions were excluded from all statistical models unless needed to generate least squares means that were important for understanding the main effects. The denominator degrees of freedom for daily activity, BR F-statistics were approximated using the Kenward–Roger’s method. For all these models, a simplified compound symmetry covariance structure was used to model the relationships between repeated observations. Least squares treatment means for all statistical models were separated using the pairwise contrasts (PDIFF, v. 9.4, SAS Inst., Inc., Cary, NC, USA). Letter assignments for differences in least square means were produced using the pdmix800.sas macro as originally described by Saxton [32].

## 3. Results

### 3.1. Climatic Data

This trial spanned two very different years climatically. The 2017 year had milder weather with minimal snow and warmer temperatures. The 2016 year received record moisture in October (119 mm). In 2016, there were seven days of measurable precipitation in October (76.5 mm), six days in November (9.7 mm), and five days in December (7.1 mm) when cattle were in the pastures. In 2017, there were zero days of measurable precipitation in October, three days in November (26.7 mm), and one day in December (0.8 mm). The minimum and maximum temperatures recorded in 2016 at the USSES weather station was −16.1 °C (7 December) and 20.6 °C (10 November), respectively, and in 2017, −13.3 °C (9 December) and 15.0 °C (28 October), respectively. At the conclusion of the trial on 12 December 2016, snow was 48 cm deep, and the temperatures were below −14 °C. The lower critical temperature (LCT) for beef cattle with a dry, heavy, winter hair coat has been defined as being −7.2 °C when no wind is present and at 15.6 °C when the haircoat is wet [33]. Considering both LCT thresholds for both dry and wet days, the cattle in 2016 were below the LCT for seven days in October, seven days in November, and ten days in December. For 2017, the cattle were below the LCT for zero days in October, seven days in November, and seven days in December. The USSES weather station was 155 m lower in elevation than at the 2016 study sites and was usually 1.1 to 1.7 °C warmer. The temperatures between the USSES weather station and the 2017 experimental pastures were comparable. A spot check with a temperature data logger (Easy Log USB Data Logger EL-USB-1-Pro, Lascar Electronics, Erie, PA, USA) at the experimental pastures on 28 November 2017 showed the high temperature to be within 0.28 °C of the temperature recorded at the USSES weather station. 

### 3.2. Forage Production and Quality

Forage production for perennial and annual grasses combined (not counting browse) over all the pastures clipped in 2016 averaged 570 ± 243 kg·ha^−1^ and 626 ± 273 kg·ha^−1^ in 2017. Assuming maximal forage intake was 1.9% of body weight (based upon forage quality) for cows averaging 612 kg at a body condition score = 5, then dry matter intake was projected to be 11.63 kg·cow^−1^. At this projected maximal forage allocation, forage utilization over all days of the study in 2016 would be projected at around 4% of the available forage on display for the 1381 ha CTRT pasture, 14% for the 359 ha CCON pasture, and 19% for the small, rotated pastures. Projected maximal utilization in 2017 at the same level of forage intake would be 25% to 26% for the continuously grazed pastures, 35% and 26% for the RTRT pastures, and 59% and 37% for the RCON pastures.

**Figure 1:**
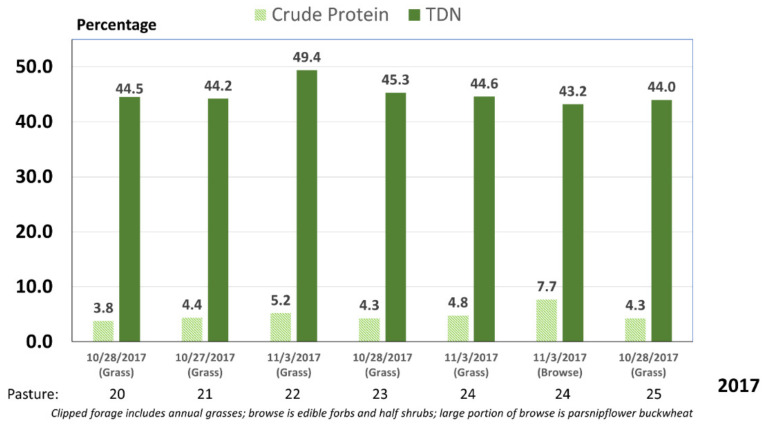
Forage quality for experimental pastures in 2017. Forage digestibility was based upon in vitro acid detergent fiber digestibility and is expressed as total digestible nutrients (TDN).

Although the forage supply was probably adequate, the analyzed forage quality did not meet nutritional requirements for the cattle in this study. Forage sampled in both 2016 (data not shown) and 2017 (Figure 1) were similar and failed to meet the total digestibility nutrient requirements necessary (52%) to prevent weight loss for nonlactating, pregnant cattle [34]. Except for a few browse samples, crude protein was also below the 7% threshold [3] needed to maintain adequate forage intake (Figure 1). 

### 3.3. Grazing Behavior

**Figure 2:**
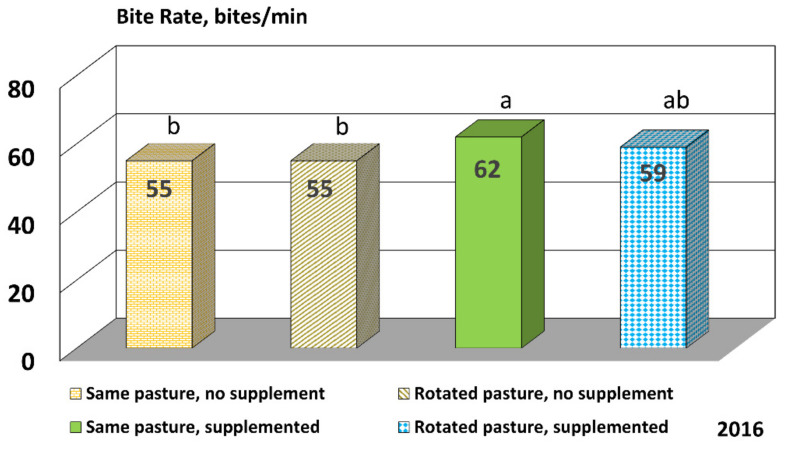
Bite rate for cattle in 2016. Cattle grazed in 4 different pasture treatments: continuously grazed with no protein supplement provided; continuously grazed with protein supplement provided; rotated through 13 pastures every 3 to 4 days with no protein supplement provided; and rotated through 13 pastures every 3 to 4 days with protein supplement provided. The 28% protein supplement was fed once per week (3.17 kg/cow) to treatment groups. Means with different letters tended to differ, *p* < 0.10.

There were no significant differences for the RFI group (*p* = 0.385)**,** pasture treatment (*p* = 0.193), or day (*p* = 0.222) for bite rate in 2016, but there was a tendency for the CTRT cattle to harvest forage faster than CCON (*p* = 0.073) and RCON (*p* = 0.065) cattle (Figure 2). There was also a significant interaction between day and pasture treatment (*p* = 0.032) in 2016, but there was no meaningful pattern among the different pasture treatments.

**Figure 3:**
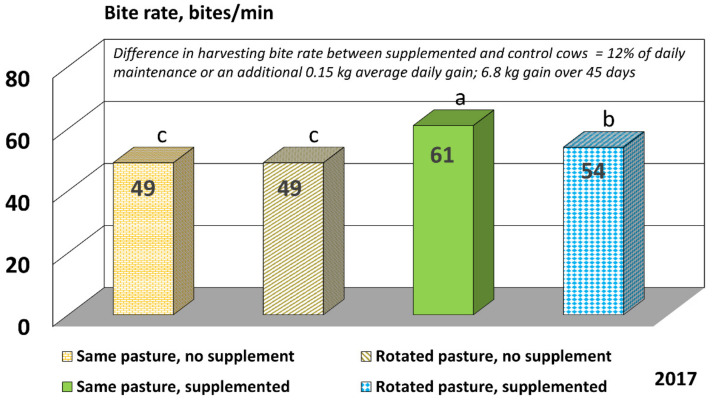
Bite rate for cattle in 2017. Cattle grazed in 4 different pasture treatments: continuously grazed with no protein supplement provided; continuously grazed with protein supplement provided; rotated to 2nd pasture after 25 days with no protein supplement provided; and rotated to 2nd pasture after 25 days with protein supplement provided. The 28% protein supplement was fed once per week (3.17 kg/cow) to the treatment groups. Means with different letters differ, *p* < 0.05.

The day × pasture treatment interaction was not significant in 2017 (*p* = 0.302), but the main effect for the RFI group tended to be significant (*p* = 0.059), and day and pasture treatment were both highly significant (*p* < 0.0001). In 2017, cattle that received no protein supplement had less (*p* < 0.05) BR than supplemented cattle (Figure 3). The CTRT cattle had the greatest (*p* < 0.001) BR in 2017. The increased BR we observed for supplemented cattle implies that animal fatigue (presumably lower energy intake and forage digestibility for non-supplemented cattle) may reduce the BR for non-supplemented cattle, or that non-supplemented cattle were engaged in searching for a higher quality diet, or both. Cattle that had a smaller harvesting rate would be taking less bites at each feeding station.

In our study, supplemented cattle could be expected to gain an additional 6.75 kg over 45 days due to their increased BR when compared to non-supplemented cattle if forage quality for supplemented cattle is affixed at 0.55 Mcal Net Energy for Gain (NE_g_)·kg^−1^ consumed forage and dry matter intake (DMI) at 0.25 g·bite^−1^ (Equation (1)). Or, considered another way, the increased BR of supplemented cattle could account for 12% of their daily maintenance requirement (estimated at 20 Mcal Metabolizable Energy (ME)·d^−1^ for 2-year-old cattle; 1.26 kg added intake·d^−1^; estimated forage quality 1.95 Mcal ME·kg forage^−1^).

(1) $$\frac{8\;bites\;}{min}\times \frac{0.25\;g\;DMI}{bite}\times \frac{60\;min}{h\;grazing}\;\times \frac{10.5\;h\;grazing}{d}\times \frac{kg}{1000\;g}\;\times \frac{0.55\;Mcal\;NEg}{kg\;DMI}\times \frac{kg\;gain}{4.63\;Mcal\;NEg}\;=\frac{0.15\;kg\;gain}{d}\times 45\;d=6.75\;kg\;gain$$

**Figure 4:**
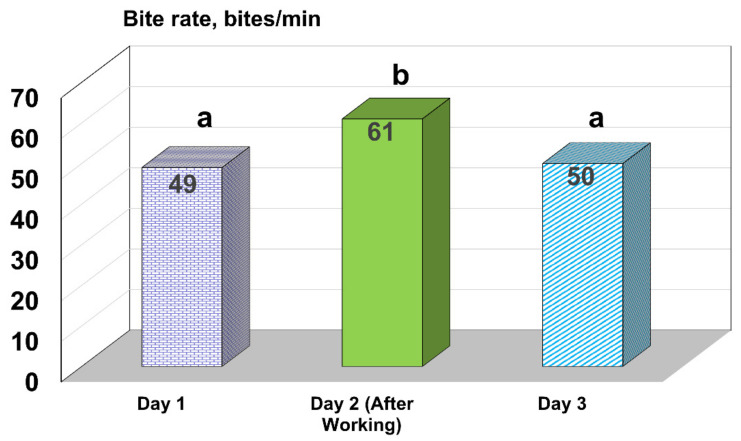
Bite rate for cattle in 2017. Day 1 was on either 30 or 31 October, Day 2 was on 21 November the day after cattle were worked in a corral to obtain weights and body condition scores. Day 3 was on 28 November. Cows increased their bite rate substantially the day following being worked in the corral. Data were recorded on 24 cows for days 1 and 2 and 23 cows on day 3. Means with different letters different, *p* < 0.001.

It is interesting to note that on the day following being worked in a corral (accompanied by removal from pastures and the opportunity to graze) in 2017, the BR increased significantly (*p* < 0.0001) when compared to other normal days not preceded by an interruption in grazing (Figure 4). Undoubtedly, this was driven by increased appetite that followed grazing removal and should be a caution for researchers on minimizing as much as possible the possibility of disturbing individual cattle behavior. 

The daily budget for how cattle partitioned their grazing and resting activities is interesting to consider. A consideration of each statistical difference for the main effect of pasture treatment for each 2 h time period within a 24 h day would be extremely tedious to explain. Therefore, the statistical differences described in Table 2 are explained for overall patterns. Generally speaking, cattle on the CCON treatment rested less during the early a.m. hours (0000 to 0800) and during the 2000 to 2200 h time period (Table 2). The cattle in this study also shifted patterns of grazing with regards to climate (Table 3). Although the grazing halters were removed on 17 November in 2016, cattle had already experienced days below the LCT 32% of the time. Over the entire grazing period, cattle in 2017 experienced days below the LCT 31% of the time, but only 4 days were associated with rain or snow compared to 18 days in 2016 (9 days during the period when collars were mounted).

To eliminate bias from the daylight length and total days of grazing for 2016 vs. 2017, Table 3 summarizes only the 9 day time period from 27 October to 4 November (preceding change in time from daylight saving to standard time) for both years. The main effect *p*-values for year for each 2 h time period with the 24 h day are shown in Table 3. In 2016, a year characterized by a more severe climate (4 days of the 9 days < LCT), cattle commenced grazing earlier (*p* < 0.0001) in the morning than they did the following year (1 day of the 9 days < LCT). Furthermore, these cattle appeared to rest more during the 0800 to 1400 time period. To summarize, cattle during the colder 2016 year engaged in more activity during the colder hours and rested more during the warmer hours of the day to help achieve thermoregulation [35].

## 4. Discussion 

### 4.1. Climatic Effects

As temperatures decrease below the LCT, each 1 °F drop in temperature increases the maintenance feed energy requirements by 1% [33]. This is reported to be linked to faster ruminal passage rates and greater rumen motility accompanied by decreased digestibility of forage as temperatures decline below the LCT [36]. Older, experienced cattle learn strategies to reduce cold stress on winter rangeland [35,37]. Younger 2-year-old cattle are still growing and lack the experience of their older herd mates, and usually lose greater weight when presented with winter grazing challenges [20].

In 2016, a year characterized by a more severe climate, the cattle commenced grazing earlier (*p* < 0.0001) in the morning than they did the following year. This conflicts with the results from Adams et al. [38], who reported that grazing began later in the day as temperatures dropped; however, the cattle in that study were subjected to much lower temperatures for longer time periods with a high temperature of 0 °C and a low of −33.9 °C. The cattle in our study in 2016 grazed more and rested less during the evening hours than they did in 2017. Additional resting during the 0800 to 1400 time period was probably related to the ability to capture solar energy for thermal warming [35]. Cattle grazing native rangelands appear to be quite adept in adapting to their environment if they were able to secure adequate feed resources.

### 4.2. Nutritional Quality and Supplementation Strategies

The nutritional quality for cattle in this study was below the maintenance requirements (Figure 1). Admittedly, cattle likely consumed a higher quality diet than what was sampled [31], somewhat mediating these negative forage quality effects.

Huston et al. [39] found that providing protein supplementation at weekly intervals was just as effective as daily or three times per week supplementation with respect to cow performance. Furthermore, they found that cows fed daily had more variable supplement intake, forage intake, and BW change than animals fed less frequently. We assume that the higher variability associated with daily supplementation is associated with disturbances to grazing activity. Wyffels et al. [40] reported that weaned heifers supplemented daily with a high-fiber protein cake (20% crude protein) from December to April spent less time grazing (6.24 h·d^−1^ vs. 6.92 h·d^−1^) and reduced the zone of herbage use when compared to heifers being provided with a self-fed high protein (62%), salt-limited supplement. Wagnon [41] reported that daily supplementation of cattle on California rangeland at 0800 h resulted in supplemented cattle grazing 2.8 h less each day when compared to non-supplemented cattle.

### 4.3. Grazing Behavior

Similar to the Sprinkle et al. [20] grazing behavior trial conducted at USSES, we found no statistical difference (*p* > 0.05) in BR between the LRFI and HRFI cattle. Lahart et al. [42] suggested that LRFI cattle have fewer grazing bouts with less aggressive harvesting rates than do HRFI cattle. Similarly, Sprinkle et al. [22] reported that LRFI lactating cattle grazing spring forage during mid-lactation had less daily grazing time than did HRFI cattle if the ambient temperatures were not elevated; however, as both forage quality and temperatures decline during late-season grazing, BR increases, particularly with advancing winter storms [20]. The increased need for diet selection by animals grazing low-quality, late-season rangeland forage may override some inherent characteristics of harvesting efficiency by LRFI vs. HRFI cattle.

In this study, we report that supplemented cattle harvested forage faster than non-supplemented cattle in 2017 (Figure 3). Previous research reported by Barton et al. [43] suggests that supplemented steers spend a greater percentage of time engaging in “intense” grazing (vs. search grazing) when compared to non-supplemented steers (93 vs. 88%). Intense grazing has been defined as an animal taking numerous bites without moving to a new feeding station whereas search grazing is defined as an animal taking a few bites at a feeding station and then moving on to the next feeding station (definitions from [44]). Krysl and Hess [44] reviewed numerous supplementation studies and concluded that providing protein supplements to cattle grazing low-quality forages increased harvest efficiency (g forage organic matter intake·kg BW^−1^·min spent grazing^−1^) from 8% to 60%. Thus, supplementing cattle with a “small package” protein supplement while they are grazing poor quality forages can decrease the energy expenditure and improve overall performance.

**Figure 5:**
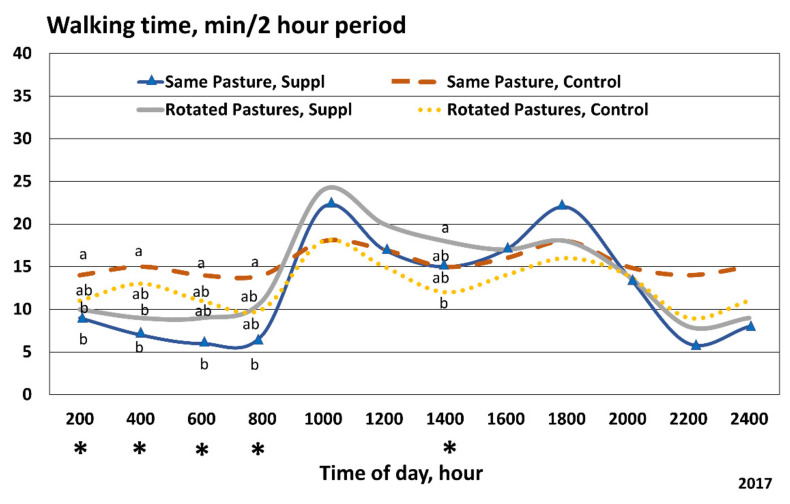
Daily walking time (minutes for every 2 h time period) for cattle grazed in 4 different pasture treatments in 2017: continuously grazed with no protein supplement provided; continuously grazed with protein supplement provided; rotated to 2nd pasture after 25 days with no protein supplement provided; and rotated to 2nd pasture after 25 days with protein supplement provided. The 28% protein supplement was fed once per week (3.17 kg/cow) to treatment groups. Means with different letters differ, *p* < 0.05 or *p* < 0.01 (also marked with *).

Cattle that stayed in the same pasture and did not receive any supplementation (CCON cattle) appeared to engage in more search grazing (less daily resting, more daily walking [23]) and altered their pattern of daily grazing (Table 2 and Figure 5). Figure 5 readily illustrates that cattle that did not have any opportunity for either increased dietary selection over time (rotational grazing) or for improved nutritional status (dietary supplementation) appeared to be more “restless” in their daily activity. Daily walking activity for the CCON cattle appeared to be uncoupled from the normal bimodal patterns of daily grazing as noted for the other supplementation treatments. It would be expected that walking activity would increase as cattle arise and start their morning grazing bout. Early morning walking activity was greater for the CCON cattle than for the CTRT cattle (*p* < 0.05) for all time periods until the morning grazing bout began. We suggest that cattle in the CCON treatment were spending more time walking, searching for better quality forage to improve their overall nutritional state.

## 5. Conclusions

Cows that were not supplemented and stayed in the same pasture through the entire grazing period engaged in more search grazing. Harvesting efficiency was greater for cattle that received protein supplementation. Cattle adapted their daily grazing patterns in response to nutritional status, grazing system, and climate to help achieve thermoregulation and nutritional homeostasis.

## Figures and Tables

**Table 1:** Protein supplement analysis.

Item	Guaranteed Analysis, (As is)	Typical Analysis, (As is)	Typical Analysis, (Dry Matter)
Dry matter, %	-	90.0	90.0
Crude protein, %	28.0	28.0	31.0
Crude fat, %	8.0	9.0	10.0
Crude fiber, %	10.0	6.2	6.9
Phosphorus, %	0.70	0.75	0.85

Bova Cubes are 100% dried distillers grains with solubles in cube form. Manufactured by Furst–McNess Company, Freeport, IL, USA. Sourced from Ord, NE, USA.

**Table 2:** Daily activity by time of day for 2-year-old cows grazing late-season rangeland ^1^.

		Pasture Treatment ^2^			
Grazing Period, h	Daily Activity, min	CCON	CTRT	RCON	RTRT
0000 to 0159	Grazing	57 ± 3.2 ^a^	52 ± 3.4 ^a^	46 ± 3.5 ^a^	47 ± 4.1 ^a^
0000 to 0159	Resting	49 ± 3.4 ^b^	60 ± 3.6 ^ab^	62 ± 3.7 ^a^	63 ± 4.3 ^ab^
					
0200 to 0359	Grazing	46 ± 3.3 ^a^	39 ± 3.5 ^a^	41 ± 3.4 ^a^	40 ± 3.6 ^a^
0200 to 0359	Resting	59 ± 4.3 ^b^	74 ± 4.6 ^a^	67 ± 4.5 ^ab^	73 ± 4.7 ^a^
					
0400 to 0559	Grazing	29 ± 4.7 ^a^	24 ± 5.0 ^a^	17 ± 5.0 ^a^	17 ± 5.0 ^a^
0400 to 0559	Resting	77 ± 5.8^a^	90 ± 6.1^a^	93 ± 6.1^a^	94 ± 6.2^a^
					
0600 to 0759	Grazing	32 ± 3.5 ^a^	28 ± 3.7 ^ab^	22 ± 3.7 ^b^	23 ± 3.7 ^ab^
0600 to 0759	Resting	73 ± 4.1 ^b^	85 ± 4.3 ^ab^	88 ± 4.3 ^a^	87 ± 4.3 ^a^
					
0800 to 0959	Grazing	87 ± 5.6 ^a^	72 ± 5.1 ^a^	84 ± 5.0 ^a^	76 ± 5.3 ^a^
0800 to 0959	Resting	29 ± 5.5 ^a^	21 ± 5.7 ^a^	16 ± 5.7 ^a^	14 ± 5.8 ^a^
					
1000 to 1159	Grazing	71 ± 2.6 ^b^	68 ± 2.8 ^b^	82 ± 2.7 ^a^	72 ± 2.8 ^b^
1000 to 1159	Resting	32 ± 2.5 ^a^	35 ± 2.6 ^a^	24 ± 2.6 ^b^	27 ± 2.7 ^ab^
					
1200 to 1359	Grazing	59 ± 2.6 ^b^	60 ± 2.7 ^b^	66 ± 2.7 ^ab^	68 ± 2.8 ^a^
1200 to 1359	Resting	45 ± 2.7 ^a^	45 ± 2.8 ^a^	42 ± 2.7 ^ab^	34 ± 2.9 ^b^
					
1400 to 1559	Grazing	61 ± 3.1 ^b^	65 ± 3.2 ^ab^	73 ± 3.2 ^a^	72 ± 3.3 ^a^
1400 to 1559	Resting	42 ± 3.5 ^a^	37 ± 3.6 ^ab^	33 ± 3.6 ^ab^	31 ± 3.7 ^b^
					
1600 to 1759	Grazing	77 ± 4.9 ^c^	81 ± 5.2 ^bc^	97 ± 5.1 ^a^	93 ± 5.2 ^ab^
1600 to 1759	Resting	25 ± 5.4 ^a^	17 ± 5.7 ^ab^	7 ± 5.7 ^b^	9 ± 5.7 ^ab^
					
1800 to 1959	Grazing	60 ± 3.6 ^a^	64 ± 3.8 ^a^	68 ± 3.8 ^a^	66 ± 3.9 ^a^
1800 to 1959	Resting	44 ± 4.0 ^a^	42 ± 4.2 ^a^	39 ± 4.2 ^a^	40 ± 4.3 ^a^
					
2000 to 2159	Grazing	30 ± 3.6 ^a^	30 ± 3.8 ^a^	18 ± 3.8 ^b^	19 ± 3.9 ^ab^
2000 to 2159	Resting	76 ± 4.8 ^b^	84 ± 5.0 ^ab^	93 ± 5.0 ^a^	93 ± 5.1 ^a^
					
2200 to 2359	Grazing	36 ± 2.7 ^b^	45 ± 2.8 ^a^	31 ± 2.8 ^b^	33 ± 3.2 ^b^
2200 to 2359	Resting	69 ± 3.0 ^ab^	66 ± 3.1 ^b^	78 ± 3.1 ^a^	78 ± 3.4 ^ab^

^1^ Daily walking data are shown in Figure 5. ^2^ CCON = continuously grazed pasture, no protein supplement (*n* = 360 d from 12 cows); CTRT = continuously grazed pasture, 3.17 kg of 28% protein supplement per cow/wk fed once a week (*n* = 326 d from 11 cows); RCON = rotational grazing, no protein supplement (*n* = 345 d from 11 cows); RTRT = rotational grazing, 3.17 kg of 28% protein supplement per cow/wk fed once a week (*n* = 295 d from 11 cows). ^a, b, c^ Means within row with differing superscripts differ, (*p* < 0.05).

**Table 3:** Daily activity by time of day and year for 2-year-old cows grazing late-season rangeland.

		Year ^1^		
Grazing Period, h	Daily Activity, min	2016	2017	*p*-Value
0000 to 0159	Grazing	51 ± 2.6	50 ± 2.6	0.8752
0000 to 0159	Resting	60 ± 2.7	58 ± 2.8	0.7288
0000 to 0159	Walking	10 ± 1.5	12 ± 1.5	0.3807
				
0200 to 0359	Grazing	45 ± 2.5	24 ± 2.6	<0.0001
0200 to 0359	Resting	66 ± 3.2	83 ± 3.3	0.0007
0200 to 0359	Walking	10 ± 2.1	13 ± 2.1	0.2715
				
0400 to 0559	Grazing	27 ± 3.9	15 ± 4.0	0.0269
0400 to 0559	Resting	84 ± 4.7	94 ± 4.8	0.1660
0400 to 0559	Walking	8 ± 2.3	11 ± 2.4	0.3229
				
0600 to 0759	Grazing	37 ± 2.5	27 ± 2.6	0.0119
0600 to 0759	Resting	72 ± 3.3	79 ± 3.4	0.1243
0600 to 0759	Walking	11 ± 1.9	13 ± 1.9	0.4406
				
0800 to 0959	Grazing	72 ± 3.9	94 ± 3.9	0.0002
0800 to 0959	Resting	25 ± 3.9	6 ± 4.0	0.0014
0800 to 0959	Walking	23 ± 2.2	20 ± 2.3	0.2976
				
1000 to 1159	Grazing	70 ± 2.8	87 ± 2.8	0.0001
1000 to 1159	Resting	31 ± 3.0	18 ± 3.1	0.0065
1000 to 1159	Walking	20 ± 1.6	15 ± 1.9	0.0618
				
1200 to 1359	Grazing	58 ± 2.0	69 ± 2.3	0.0016
1200 to 1359	Resting	44 ± 2.1	39 ± 2.4	0.0917
1200 to 1359	Walking	18 ± 1.1	13 ± 1.1	0.0018
				
1400 to 1559	Grazing	70 ± 3.2	68 ± 3.3	0.6569
1400 to 1559	Resting	32 ± 3.8	38 ± 3.9	0.2357
1400 to 1559	Walking	18 ± 1.2	14 ± 1.2	0.0103
				
1600 to 1759	Grazing	77 ± 3.9	97 ± 4.0	0.0010
1600 to 1759	Resting	20 ± 4.2	7 ± 4.3	0.0374
1600 to 1759	Walking	16 ± 2.4	21 ± 2.3	0.0394
				
1800 to 1959	Grazing	70 ± 2.6	63 ± 2.6	0.0610
1800 to 1959	Resting	35 ± 2.8	42 ± 2.9	0.0626
1800 to 1959	Walking	15 ± 1.2	15 ± 1.3	0.8032
				
2000 to 2159	Grazing	32 ± 3.4	14 ± 3.4	0.0006
2000 to 2159	Resting	78 ± 4.3	94 ± 4.4	0.0102
2000 to 2159	Walking	10 ± 2.6	11 ± 2.7	0.6684
				
2200 to 2359	Grazing	38 ± 2.4	38 ± 2.5	0.9271
2200 to 2359	Resting	71 ± 3.0	71 ± 3.0	0.9033
2200 to 2359	Walking	11 ± 1.9	11 ± 1.9	0.8612

^1^ For data collected from 27 October to 4 November; 2016 had *n* = 191 d from 23 cows and 2017 had *n* = 189 d from 22 cows.

## Data Availability

The data presented in this study are available on request from the corresponding author. The data are not publicly available due to very large datasets exceeding 100 MB cumulative size.

## Supplementary Materials

Information on using accelerometers for determining grazing behavior and in processing data are fully described in Sprinkle et al. (2021b; citation [24]). Additional resources are available at the shared website folder (Available online: https://app.box.com/s/ayzk1e2zskinotjyjypv1u4whluc7roa (accessed on 8 November 2021)) containing example data, programming code, spreadsheets, hands on training exercises, and an instruction manual.
